# Clinical and pathological characteristics of ANA/anti-dsDNA positive patients with antineutrophil cytoplasmic autoantibody-associated vasculitis

**DOI:** 10.1007/s00296-020-04704-3

**Published:** 2020-09-22

**Authors:** Xiaohong Zhao, Qiong Wen, Yagui Qiu, Fengxian Huang

**Affiliations:** 1 Department of Nephrology, The First Affiliated Hospital, Sun Yat-sen University, Key Laboratory of Nephrology, National Health Commission and Guangdong Province, 58th Zhongshan Road II, Guangzhou, 510080 China; 2 Department of Nephrology, Shenzhen Key Laboratory of Nephrology Diseases, Shenzhen People’s Hospital (The Second Clinical Medical College, Jinan University; The First Affiliated Hospital), Southern University of Science and Technology, Shenzhen, Guangdong, 518020 China

**Keywords:** Antineutrophil cytoplasmic autoantibodies (ANCA), Associated vasculitis (AAV), Clinicopathological characteristics, Anti-nuclear antibody (ANA), Anti-double stranded DNA antibodies (anti-dsDNA)

## Abstract

Antineutrophil cytoplasmic autoantibodies (ANCA) associated vasculitis (AAV) consists of a group of systemic autoimmune diseases. The roles of serum anti-nuclear antibodies (ANA) and anti-double-stranded DNA (anti-dsDNA) antibodies in AAV patients remain unknown. This study investigated the prevalence of serum ANAs and anti-dsDNA antibodies in AAV patients and characterized the clinical and pathological features of these patients. A total of 218 AAV patients were enrolled. Clinical and pathological data of patients were analyzed retrospectively. Of the 218 AAV patients, 109 (50.0%) were positive for ANA, 45 (20.6%) were positive for anti-dsDNA, and 43 (19.7%) were positive for both. The AAV patients with ANA had severer kidney damage and more chronic renal histopathological changes compared to those who were negative for ANA. Specifically, patients positive for ANA had more hypertension, higher levels of urea nitrogen and serum creatinine, lower estimated glomerular filtration rate (eGFR), more end-stage renal disease (ESRD), severer proteinuria, glomerular sclerosis, tubular interstitial fibrosis and tubular atrophy, and were more likely to receive renal biopsies compared to ANA negative patients. The study found ANA and anti-dsDNA in AVV patients were not rare, ANA-positive AAV patients had severer kidney damage and more chronic renal histopathological changes compared to ANA-negative AAV patients. Renal biopsy is strongly recommended for differential diagnosis in such cases.

## Introduction

Antineutrophil cytoplasmic autoantibodies (ANCA) associated vasculitis (AAV) is a group of autoimmune diseases that share features of necrotizing small vessel vasculitis and various frequency of ANCA-association, which includes microscopic polyangiitis (MPA), granulomatosis with polyangiitis (GPA, also called Wegener’s granulomatosis (WG)), and eosinophilic granulomatosis with polyangiitis [EGPA, also called Churg-Strauss syndrome (CSS)] [1, 2]. AAV is a multisystem disease and its clinical manifestations vary, which can lead to misdiagnosis or mistreatment. ANCA is a specific serological marker of AAV. Renal involvement is particularly important in AAV, usually manifesting as rapidly progressive glomerulonephritis. Generally, the classical renal involvement of ANCA-associated small-vessel vasculitis is pauci-immune necrotizing and crescentic glomerulonephritis. Although the pathogenesis of AAV has not been extensively studied, in vitro and in vivo evidence suggests that ANCA plays a central role in mediating small vessel vasculitis. Retrospective study reported that ANCA in lupus nephritis patients was not rare, and patients with ANCA presented with more severe clinicopathologic injuries [3].

It is well-established that antibodies, such as anti-nuclear antibody (ANA) and anti-double-stranded DNA (anti-dsDNA) antibodies, are of diagnostic significance in systemic lupus erythematosus (SLE), which may cause renal damage via a variety of mechanisms [4–8]. ANA may cause organ/tissue injuries by mechanisms such as immune complex deposition, cytokine stimulation, or receptor binding. Anti-dsDNA antibodies may promote nephritis by immune complex deposition and direct binding to sites in the glomerulus. Previous studies have shown that 18–66% of AAV patients tested positively for serum ANA, but the potential clinical and pathological significance of this observation is not clear [9, 10]. Anti-dsDNA positive in AAV patients have not yet been reported in the current literature. However, we observed that AAV patients were positive for ANA and/or anti-dsDNA. Furthermore, we retrospectively analyzed the clinical and pathological characteristics of ANA and anti-dsDNA positive AAV patients.

## Methods

This is a retrospective cohort study aimed at analyzing the clinical and pathological characteristics of ANA and anti-dsDNA positive AAV patients. This study was approved by the Ethics Committee of The First Affiliated Hospital, Sun Yat-sen University. All procedures performed in studies involving human participants were in accordance with the ethical standards of the institutional and national research committee and with the 1964 Helsinki declaration and its later amendments or comparable ethical standards. Written informed consent was obtained from all individual participants included in the study.

### Patients

A total of 218 AAV patients (age ≥ 14 year) diagnosed in the First Affiliated Hospital of Sun Yat-sen University between January 1, 2001 and October 31, 2014 were enrolled in this study. All patients met the criteria of the Chapel Hill consensus conference definition (for WG, MPA, or CSS) and American College of Rheumatology (ACR) classification criteria (for WG or CSS). Patients with SLE, Henoch-Scholein purpura, rheumatoid arthritis, or propylthiouracil-induced vasculitis were excluded. Patients who re-admitted after treatment were excluded.

### Data collection

Clinical, biological, and immunological data were collected at AAV diagnosis. Perinuclear staining antineutrophil cytoplasmic antoantibody (pANCA) and cytoplamic staining antineutrophil cytoplasmic antoantibody (cANCA) were evaluated using the indirect immunofluorescence assay. Anti-myeloperoxidase (MPO), anti-proteinase 3 (PR3), anti-glomerular basement membrane (GBM) were detected using enzyme-linked immunosorbent assay (ELISA). Serum antinuclear antibodies and anti-dsDNA antibodies were detected using the indirect immunofluorescence assay. Antiextractable nuclear antigen antibodies, including anti-Sm, anti-SSA, anti-SSB were detected using the immunodotting assay. Anticardiolipin antibodies were detected using ELISA, according to the protocols provided by the manufacturer. Extra-renal clinical manifestations included fever, fatigue, weight loss, rash, arthralgia or muscle pain, serous cavity effusion, hemoptysis, gastrointestinal involvement, and neurological disorders. Gastrointestinal involvement included pain and/or gastrointestinal bleeding. Neurological disorders included seizures or multifocal neural deficit (mononeuritis multiplex). Renal involvement was defined by the presence of hematuria, proteinuria, or both upon urinalysis with or without renal insufficiency. Estimated glomerular filtration rate (eGFR) was calculated using the Chronic Kidney Disease Epidemiology Collaboration equation [11].

### Pathological examination

Of the 108 AAV patients who received a renal biopsy, complete renal histopathological data were available in 93 cases. (The renal histopathological data of the remaining 15 AAV patients were incomplete for various reasons). For classification of glomerulonephritis, kidney specimens had to have a minimum of ten glomeruli visible under light microscopy. Renal specimens were examined using immunofluorescence, as well as light and electron microscopy.

### Statistical analysis

All statistical analyses were conducted using SPSS V. 22.0. Data are shown as mean with 95% confidence interval (CI) or median when appropriate. The 1-sample *K*–*S* test was used to determine the normality of the data distribution. A two-sample Student *t* test was used to compare quantitative variables (mean ± standard deviation (SD)], and the Fisher exact test was used to compare distributions of categorical variables between groups. Comparisons between groups were made with ANOVA or Kruskal–Wallis test. A *P* value < 0.05 was considered statistically significant.

## Results

### Demographic features

Of the 218 AAV patients in this study, half were male and half were female. The median age was 56 years (range 14–81 years), and 61.0% patients were > 50 years old. There was no significant difference in age between males and females (*P* > 0.05). The number of patients newly diagnosed with AAV increased with each study year.

### ANCA patterns

pANCA or anti- MPO was detected in 166 of 218 (76.1%) patients, whereas cytoplasmic (cANCA) or anti-proteinase 3 (PR3) was detected in 19 of 218 (8.7%) patients. Nine patients were atypical pANCA with perinuclear fluorescence and where the antigen was not MPO. Two patients were atypical C-ANCA with flat cytoplasmic fluorescence and where the antigen was not PR3. Twenty-two (10.1%) patients were both pANCA-MPO and cANCA-PR3 positive, and 11 (4.8%) patients were ANCA negative. Of the 218 AAV patients, 187 patients (85.8%) were MPA, 23 patients (10.6%) were GPA, and 8 (3.7%) patients were EGPA.

### Epidemiological features

In the present study, we detected various antibodies, including ANA, anti-dsDNA, anti-GBM, Coombs, anti-SSA, anti-SSB, and anti-cardiolipin antibodies, in the sera of AAV patients (Table 1). Of the 218 AAV patients, 109 (50.0%) were ANA positive and 45 (20.6%) were anti-dsDNA positive. Of the 109 ANA positive patients, 63 were female and 46 were male (*P* = 0.021). Of the 45 anti-dsDNA positive patients, 29 were female and 16 were male (*P* = 0.030). These data indicate that ANA and anti-dsDNA are more common in female AAV patients.

**Table 1:** Laboratory findings in AAV patients

Items	Cases	Rate (%)
ANA (+)	109/218	50.0%
Anti-dsDNA (+)	45/218	20.6%
Anti-Smith (+)	1/218	0.5%
Anti-DNP (+)	59/218	27.1%
Anti-RNP (+)	3/218	1.4%
Anti-SSA/Roanti-SSB/La	19/218	8.7%
SSA (+) SSB (+)	6/218	2.8%
SSA (+) SSB (−)	11/218	5.0%
SSA (−) SSB ( +)	2/218	0.9%
Anticardiolipin antibody	61/229	26.6%
Anti-GBM antibodies	3/126	2.4%
Coombs (+)	10/56	23.8%
C3 (< 0.79)	48/218	23.2%
C4 (< 0.17)	31/218	15.0%
ESR (> 20 mm/h)	149/168	88.7%
CRP (> 3.0 mg/L)	161/208	77.4%

### Renal and extra-renal involvement

A total of 182 cases (83.5%) had renal involvement, 34.4% (75/218) patients developed ESRD at the time of AAV diagnosis. Of these, 2.6% presented with nephrotic syndrome, which was relatively infrequent. To varying degrees, the lungs (71/218, 32.6%), digestive system (78/218, 35.8%), nervous system (41/218, 18.8%), eyes (7/218, 3.2%), ears (7/218, 3.2%), noses (4/218, 1.8%), and vocal cords (3/218, 1.4%) were affected in the AAV patients.

### Clinical characteristics

**Fig. 1:**
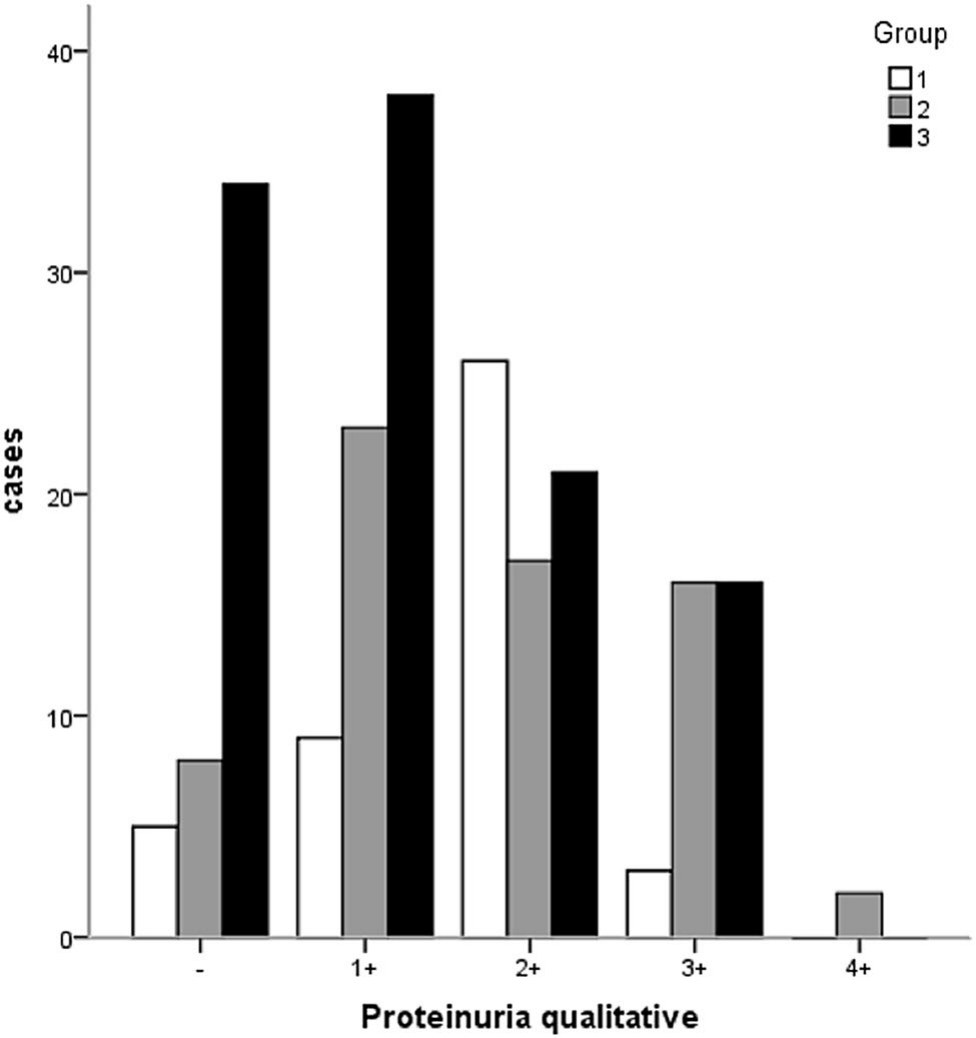
Patients positive for ANA in the serum (group 1 and group 2) presented with severer proteinuria clinically. (− ~ 4 +) indicates the degree of proteinuria

To identify the potential roles of ANA and anti-dsDNA in AAV patients, we divided the 218 patients into three groups and compared their clinical characteristics. In group 1, AAV patients were both ANA and anti-dsDNA positive. In group 2, AAV patients were ANA positive but anti-dsDNA negative. In group 3, AAV patients were ANA negative. As shown in Table 2, there were no differences in extra-renal clinical manifestations between groups. However, the patients who were ANA positive had severer kidney damage compared to those who were negative for ANA (Table 3). ANA positive patients (group 1 and group 2) had higher percentages of hypertension, higher levels of urea nitrogen and serum creatinine, lower eGFR, higher frequencies of ESRD, and severer proteinuria (Fig. 1). These patients were also more likely to receive renal biopsies compared to those who were ANA negative (group 3). However, no statistical differences were observed in the above mentioned renal clinical manifestations between group 1 and group 2.

**Table 2:** Comparison of extra-renal clinical manifestations in the three groups

Extra-renal clinical manifestation	0verall	ANA ( +) anti-dsDNA ( +)	ANA ( +) anti-dsDNA (−)	ANA ( −) anti-dsDNA(-)/anti-dsDNA( +)*	*P*
	(*n* = 218)	(*n* = 43)	(*n* = 66)	(*n* = 109)	
Age (year)	51.97 ± 16.01	53.56 ± 13.92	53.30 ± 16.36	50.54 ± 16.56	0.419
Fever *n* (%)	73 (33.49)	11(25.58)	22 (33.33)	40 (36.70)	0.425
Fatigue *n* (%)	81(37.16)	20 (46.51)	22 (33.33)	39 (35.78)	0.348
Weight loss *n* (%)	76 (34.86)	18 (41.86)	23 (34.85)	35 (32.11)	0.524
Rash *n* (%)	17 (7.80)	4 (9.30)	3 (4.55)	10 (9.17)	0.498
Arthralgia/Muscle pain *n* (%)	44 (20.18)	10 (23.26)	9 (13.64)	25 (22.94)	0.284
Serous cavity effusion *n* (%)	60(27.52)	11 (35.58)	22 (33.33)	27 (24.77)	0.447
Haemoptysis *n* (%)	29 (13.30)	7 (16.28)	11 (16.67)	11 (10.09)	0.377
Gastrointestinal involvement *n* (%)	78 (35.78)	16 (37.21)	25 (37.88)	37 (33.94)	0.850
Neurological disorders *n* (%)	41 (18.81)	7 (16.28)	16 (24.24)	18 (16.51)	0.400

*Two patients were ANA negative and low liter dsDNA positive (probably false positive). These two patients divided into ANA negative group in the data analysis

**Table 3:** Comparison of renal clinical manifestations in the three groups

Renal clinical manifestation		Overall	ANA (+) anti-dsDNA (+)	ANA (+) anti-dsDNA( −)	ANA (−) anti-dsDNA (−)/anti-dsDNA (+)	*P*
		(*n* = 218)	(*n* = 43)	(*n* = 66)	(*n* = 109)	
Edema *n* (%)		76 (34.86)	19 (44.19)	26 (39.39)	31 (28.44)	0.121
Oliguria/anuria *n* (%)		48 (22.02)	10 (23.26)	17 (25.76)	21 (19.27)	0.590
Gross hematuria *n* (%)		22 (10.09)	8 (18.60)	6 (9.09)	8 (7.34)	0.110
Hypertension *n* (%)		108 (49.54)	27 (62.79) ^**#**^	37 (56.06)^**&**^	44 (40.37)	**0.020**
Urea nitrogen (mmol/L)		14.28 ± 10.08	16.29 ± 9.36^**#**^	16.57 ± 10.46^**&**^	12.10 ± 9.73	**0.006**
Serum creatinine (umol/L)		311.32 ± 290.93	371.09 ± 256.61^**#**^	372.83 ± 297.43^**&**^	250.50 ± 289.38	**0.008**
eGFR (mL/min/1.73 m^2^)		47.53 ± 43.36	28.13 ± 30.70^**#**^	35.05 ± 37.38^**&**^	62.75 ± 45.74	**0.000**
ESRD *n* (%)		75 (34.40)	20 (46.51)^#^	28 (42.42)^**&**^	27 (24.77)	**0.010**
Hematuria *n* (%)		138 (63.30)	31 (72.09)	43(65.15)	64 (58.72)	0.169
Urine erythrocyte under microscope	≤ 3/HP	80 (36.70)	12 (27.91)	23 (34.85)	45(41.28)	
	3–5/HP	6 (2.75)	1 (2.33)	0 (0)	5 (45.87)	
	+	45 (20.64)	9 (20.93)	14 (21.21)	22 (20.18)	
	2 +	41 (18.81)	12 (27.91)	12 (18.18)	17 (15.60)	
	3 +	34 (15.60)	6 (13.95)	12 (18.18)	16 (14.68)	
	4 +	12 (5.50)	3 (6.98)	5 (7.58)	4 (36.70)	
Proteinuria *n* (%)		171(78.44)	38 (88.37)^**#**^	58 (87.88)^**&**^	75 (68.81)	**0.001**
Qualitative	−	47 (21.56)	5 (11.63)	8 (12.12)	34(31.19)	
	1 +	70(32.11)	9 (4.13)	23 (34.85)	38 (34.86)	
	2 +	64(29.36)	26 (60.47)	17 (25.76)	21 (19.27)	
	3 +	35(16.06)	3 (6.98)	16 (24.24)	16 (14.68)	
	4 +	2 (0.92)	0 (0)	2 (3.03)	0 (0)	
Renal biopsies *n* (%)		108 (49.54)	35 (81.40)^*#^	32 (48.48)	41 (37.61^**)**^	**0.000**

*G1 compared with G2: *P* < 0.05; ^**#**^G1 compared with G3: *P* < 0.05; and G2 compared with G3: *P* < 0.05

*P* value:

Hypertension: G1 compared with G2: *P* = 0.485, G1 compared with G3: *P* = 0.013, G2 compared with G3: *P* = 0.044

Urea nitrogen: G1 compared with G2: *P* = 0.873

Cr: G1 compared with G2: *P* = 0.973

eGFR: G1 compared with G2: *P* = 0.340

ESRD: G1 compared with G2: *P* = 0.674, G1 compared with G3: *P* = 0.009, G2 compared with G3: *P* = 0.015

Renal biopsies: G1 compared with G2: *P* = 0.001, G1 compared with G3: *P* = 0.000, G2 compared with G3:* P* = 0.158

Proteinuria: G1 compared with G2: *P* = 0.938, G1 compared with G3: *P* = 0.013, G2 compared with G3: *P* = 0.004

### Renal histopathological characteristics

Complete renal histopathological data were available for 93 AAV patients; 76.3% (71/93) had pauci-immune necrotizing and crescentic glomerulonephritis, and 11.8% (11/93) had proliferative sclerotic nephritis / sclerotic nephritis. Furthermore, some patients presented with features of membranous nephropathy (4/93), Immunoglobin A (IgA) nephropathy (3/93), focal segmental glomerulosclerosis (1/93), and type I crescentic glomerulonephritis (1/93). Some patients showed immunoglobulin deposition, as evidenced by immunofluorescence imaging. The average percentage of glomerular sclerosis and crescent were each 34.5%, respectively. Loop necrosis (49.5%) and saccule adhesion (41.9%) were common, while tubulointerstitial lesions (89.3%) were highly prevalent.

Based on the serum ANA testing results, we divided 93 patients into two groups and compared their renal histopathological features. The AAV patients who were serum ANA positive had more chronic renal histopathological changes compared to those who were ANA negative (Table 4). Specifically, the ANA positive patients had more glomerular sclerosis (*P* = 0.024), tubular interstitial fibrosis (*P* = 0.004), and tubular atrophy (*P* = 0.021) compared to the ANA negative patients.

**Table 4:** Comparison of renal histopathological features between ANA positive and negative patients

	Overall	ANA ( +)	ANA ( −)	*P*
	(*n* = 93)	(*n* = 56)	(*n* = 37)	
Crescentic glomerulonephritis *n* (%)	71 (76.34)	45 (80.36)	26 (70.27)	0.263
Glomerular sclerosis (%)	33.01 ± 27.43	38.16 ± 28.25	25.23 ± 24.51	0.024
Focal segmental glomerular sclerosis (%)	2.39 ± 5.33	2.01 ± 3.90	2.97 ± 6.99	0.996
Crescents formation (%)	34.50 ± 23.63	32.92 ± 21.94	36.89 ± 26.22	0.520
Cellular crescents (%)	6.56 ± 14.89	7.11 ± 16.34	6.36 ± 5.71	0.891
Mesangial cells and matrix hyperplasia *n* (%)	56 (60.22)	31 (55.36)	25 (67.57)	0.354
Little	37	25	12	
Mild	43	23	20	
Medium	12	8	4	
Severe	1	0	1	
Endothelial proliferation *n* (%)	15 (16.13)	6 (10.71)	9 (24.32)	0.081
Leukocytes infiltration *n* (%)	8 (91.40)	4 (71.43)	4 (10.81)	0.709
Loop necrosis *n* (%)	46 (49.46)	29 (51.79)	17 (45.95)	0.685
None	47	27	20	
Mild	27	17	10	
Medium	12	9	3	
Severe	7	3	4	
Balloon adhesion *n* (%)	39 (41.94)	22 (39.29)	17 (45.95)	0.524
Interstitial inflammatory infiltration *n* (%)	89 (95.70)	53 (94.64)	36 (97.30)	0.555
None	4	3	1	
Focal	23	11	12	
Multifocal	39	26	13	
Large	9	4	5	
Diffuse	18	12	6	
Tubularinterstitial fibrosis *n* (%)	80 (86.02)	52 (92.86)	28 (75.68)	0.004
None	13	4	9	
Focal	23	11	12	
Multifocal	39	28	11	
Large	11	7	4	
Diffuse	7	6	1	
Interstitial edema *n* (%)	27 (29.03)	16 (28.57)	11 (29.73)	0.904
Tubular atrophy *n* (%)	83 (89.25)	52 (92.86)	31 (83.78)	0.021
None	10	4	6	
Focal	18	6	12	
Multifocal	38	28	10	
Large	8	5	3	
Diffuse	19	13	6	
Arterial wall thickening *n* (%)	55 (59.14)	32 (57.14)	23 (62.16)	0.630
Vascular hyaline degeneration *n* (%)	27 (29.03)	14 (25.00)	13 (35.14)	0.292

## Discussion

An increasing number of AAV patients have been diagnosed in recent years due to increased awareness and improvement in diagnostic techniques. Our study found that elderly individuals are more susceptible to AAV, consistent with previous reports [12, 13].

In this study, pANCA/MPO positive patients were more common than cANCA /PR3 positive AAV patients, which differs from previous studies that included white populations. Eleven (4.8%) patients were ANCA negative. The number of ANCA-negative AAV patients might be an underestimation since this is difficult to diagnose. Pathological examination is essential for definitively diagnosing AAV under such circumstances.

AAV is characterized by multisystem injuries and diverse clinical manifestations. Non-specific symptoms such as fever, fatigue, significant weight loss, and serious cavity effusion are common. In our study, 83.5% cases had renal involvement. The kidney is the most vulnerable organ in AAV patients. The prevalence of ear-nose-throat involvement and ocular involvement reported by a single-center retrospective study in Spain was 31.4% and 19.5%, respectively [13, 14]. However, in our study, ear-nose-throat and ocular involvement were less common. The difference may be attributed to geographical, environmental, and genetic differences, which require further study.

In AAV patients in this study, various autoimmune antibodies, such as ANA, anti-dsDNA, anti-GBM, Coombs, anti-SSA, anti-SSB, and anti-cardiolipin antibodies, were found to be positive to varying degrees. It is worth noting that ANA and anti-dsDNA positive AAV patients are not rare. It has been documented in previous studies that 18–66% of AAV patients are ANA positive, but the clinical significance remains unknown [8–10]. As far as we know, AAV patients who are positive for anti-dsDNA have not been reported. In this study, we discovered that 109 of the 218 (50.0%) patients were ANA positive. Furthermore, we are the first to report that 20.6% were anti-dsDNA positive, and 19.7% were both ANA and anti-dsDNA positive among our cohort of AAV patients.

It has been suggested that ANA and anti-dsDNA may cause renal damage via several mechanisms [4–6, 8]. Whether AAV patients who are ANA and anti-dsDNA positive have severer diseases compared to those who are negative was unclear. Therefore, we retrospectively analyzed the clinical and pathological characteristics of patients who were ANA and anti-dsDNA positive. As a result, we found that the AAV patients positive for ANA had severer kidney damage, higher percentages of hypertension, higher levels of urea nitrogen and serum creatinine, lower eGFR, higher prevalence of ESRD, severer proteinuria, and were more likely to receive renal biopsies compared to ANA negative patients. Patients positive for both ANA and dsDNA had higher percentages of hypertension, lower eGFR, higher prevalence of ESRD, and were more likely to receive renal biopsies compared to ANA positive patients. However, there were no statistical differences between these groups, which might be attributed to the limited number of cases in this study. These results suggest that ANA and anti-dsDNA aggravate impaired renal function, which is consistent with the finding that antibodies such as ANA and anti-dsDNA can cause renal damage. Nevertheless, further research is necessary to understand the underlying mechanisms.

The most common renal histopathological changes in the AAV patients in this study were pauci-immune necrotizing and crescentic glomerulonephritis. However, a few AAV patients also presented with features of membranous nephropathy, IgA nephropathy, focal segmental glomerulosclerosis, and type I, crescentic glomerulonephritis. Some AAV patients showed immune complex deposition in the renal biopsies. The above findings are consistent with previous reports [13, 15], but the composition and localization of the deposits in AAV patients have not been widely studied and their potential pathological and clinical significance remain unknown. A small but significant percentage (11.8%) of patients might have a more indolent course and present at diagnosis, with chronic pathological changes such as proliferative sclerotic nephritis and sclerotic nephritis.

By comparing the renal histopathological features, we found that ANA-positive AAV patients had more chronic renal histopathological changes compared to ANA-negative AAV patients. Moreover, 43 (19.7%) AAV patients were both ANA and anti-dsDNA positive, of which 38 (88.4%) cases had simultaneous anemia and kidney damage. Patients who were both ANA and anti-dsDNA positive who also had anemia and kidney damage that satisfied the diagnostic criteria of SLE [7]. Both AAV and SLE are autoimmune diseases involving multiple systems. It is difficult to distinguish them only through clinical manifestations in some cases, especially when the sera of patients tested positive for ANA, anti-dsDNA, and ANCA. 38 AAV patients with both ANA and anti-dsDNA positive had pauci-immune necrotizing and crescentic glomerulonephritis in renal histopathology. For those patients without renal pathological examination, it is possible that a certain proportion of patients could actually be lupus having ANCA antibodies. Under such circumstances, renal biopsy plays a key role in the differential diagnosis. The most common renal histopathological changes of AAV patients are pauci-immune necrotizing and crescentic glomerulonephritis, while lupus nephritis is immune-complex-mediated glomerulonephritis with a full-house nephropathy pattern on immunofluorescence. Without pathological data, there is no specific identification standard. Thus, Renal biopsy is recommended to differentiate between AAV and SLE when possible.

Our study has several limitations. The major limitation of this study was that only 93 AAV patients of total 218 patients had complete renal histopathological data. And these data could not be supplemented because this study was retrospective. Due to lack of renal pathology, it was hard to distinguish between AAV with ANA antibodies positive between lupus with ANCA antibodies positive. In addition, 34.4% (75/218) patients developed ESRD at the time of AAV diagnosis, some patients might develop ANA positive in the background of ESRD. Besides, other factors such as infection, tumor, together with ESRD might result in a higher positive rate of ANA antibodies. Finally, 218 AAV patients from 2001 to 2014 were enrolled in this study, quite a few patients lost to follow up, as a result, no prognosis analysis was made.

In conclusion, the present study showed that AAV patients might test positive for both ANA and anti-dsDNA. ANA positive AAV patients had severer kidney damage and more chronic renal histopathological changes compared to ANA negative patients. More importantly, most of the patients who were positive for both ANA and anti-dsDNA had anemia and kidney damage. Renal biopsy is strongly recommended to distinguish between AAV and SLE. However, further studies are necessary to investigate the role of ANA and anti-dsDNA in AAV patients.

## References

[1] Jennette JC, Falk RJ, Andrassy K (1994). Nomenclature of systemic vasculitides. Proposal of an international consensus conference. Arthritis Rheum.

[2] Jennette JC, Falk RJ, Bacon PA (2013). 2012 revised International Chapel Hill consensus conference nomenclature of vasculitides. Arthritis Rheum.

[3] Wang Y, Huang X, Cai J (2016). Clinicopathologic characteristics and outcomes of lupus nephritis with antineutrophil cytoplasmic antibody: a retrospective study. Medicine.

[4] Pinnas JL, Fenster PE (1977). ANA, DNA, and SLE. Arizona Med.

[5] Bizzaro N, Villalta D (2001). The predictive value of ANA and anti-dsDNA antibodies for flares in SLE. Rheumatology.

[6] Dema B, Charles N (2016). Autoantibodies in SLE: specificities.

[7] Aringer M, Costenbader K, Daikh D (2019). 2019 European league against rheumatism/American College of Rheumatology classification criteria for systemic lupus erythematosus. Arthritis Rheum (Hoboken, NJ).

[8] Rekvig OP (2015). Anti-dsDNA antibodies as a classification criterion and a diagnostic marker for systemic lupus erythematosus: critical remarks. Clin Exp Immunol.

[9] Savige JA, Chang L, Wilson D, Buchanan RR (1996). Autoantibodies and target antigens in antineutrophil cytoplasmic antibody (ANCA)-associated vasculitides. Rheumatol Int.

[10] Lenert P, Icardi M, Dahmoush L (2013). ANA (+) ANCA (+) systemic vasculitis associated with the use of minocycline: case-based review. Clin Rheumatol.

[11] Levey AS, Stevens LA, Schmid CH (2009). A new equation to estimate glomerular filtration rate. Ann Intern Med.

[12] Jennette JC, Nachman PH (2017). ANCA glomerulonephritis and vasculitis. Clini J Am Soc Nephrol CJASN.

[13] Chen M, Yu F, Zhang Y, Zhao MH (2005). Clinical [corrected] and pathological characteristics of Chinese patients with antineutrophil cytoplasmic autoantibody associated systemic vasculitides: a study of 426 patients from a single centre. Postgrad Med J.

[14] Tidman M, Olander R, Svalander C, Danielsson D (1998). Patients hospitalized because of small vessel vasculitides with renal involvement in the period 1975–95: organ involvement, anti-neutrophil cytoplasmic antibodies patterns, seasonal attack rates and fluctuation of annual frequencies. J Intern Med.

[15] Yu F, Chen M, Wang SX, Zou WZ, Zhao MH, Wang HY (2007). Clinical and pathological characteristics and outcomes of Chinese patients with primary anti-neutrophil cytoplasmic antibodies-associated systemic vasculitis with immune complex deposition in kidney. Nephrology.

